# The Association between Sleep and Theory of Mind in School Aged Children with ADHD

**DOI:** 10.3390/medsci5030018

**Published:** 2017-08-21

**Authors:** Rackeb Tesfaye, Reut Gruber

**Affiliations:** Department of Psychiatry, McGill University and Attention, Behavior and Sleep Lab, Douglas Mental Health University Institute, Montreal, QC H4H 1R3, Canada; rackeb.tesfaye@mail.mcgill.ca

**Keywords:** Theory of Mind, sleep, attention deficit hyperactivity disorder, executive functions, emotional information processing cognition, social functioning

## Abstract

Theory of Mind (ToM) is defined as the ability to infer a range of internal mental states of others, including beliefs, intentions, desires, and emotions. These abilities are associated with children’s ability to socialize effectively with peers. ToM impairments are associated with peer rejection and psychiatric disorders such as Attention-Deficit/Hyperactivity Disorder (ADHD). Previous studies have found poor sleep negatively impacts executive functioning (EF) and emotional information processing, which are essential for the effective use of ToM. Youth with ADHD have EF deficits and sleep problems. However, the relationship between sleep, executive functioning, and ToM in children with ADHD has not been studied. In this review, we propose that the poor social and interpersonal skills characterizing individuals with ADHD could be explained by the impact of poor sleep on the emotional and cognitive mechanisms underlying ToM.

## 1. Introduction

Theory of Mind (ToM) is defined as the ability to infer a range of internal mental states including beliefs, intentions, desire, and emotions [1,2]. It is central to the development of social cognition in children, referring to the psychological processes needed for an individual to integrate and be a part of a social group. Children’s capacity to successfully use ToM has been shown to be a positive predictor of their ability to socialize effectively [3,4,5]. ToM impairments are associated with poor social interactions [6,7] and various psychiatric [8,9,10] and neuro-developmental disorders [11]. Very little is known about the factors or mechanisms that influence the development of successful ToM in school aged children. Previous studies have found poor or insufficient sleep negatively impacts executive functioning and emotional functioning. Both of these impairments are associated with deficits in ToM. However, the relationship between sleep and ToM has not been studied. Youth with Attention-Deficit/Hyperactivity Disorder (ADHD) experience lower sleep quality and duration compared to their typically developing peers [12,13]. We propose that poor and insufficient sleep found in 50%–80% of children with ADHD contributes to the documented social impairments found in youth with ADHD. Examining the relationship between sleep and social impairments in youth with ADHD is of particular importance. First, ADHD is one of the most common neuro-developmental disorders found in childhood, with approximately 5% of school aged children diagnosed [14] and higher estimated rates found in various United States community samples [15]. Second, youth with ADHD have documented social impairments, including greater peer rejection [16,17], impaired expression of empathy [18], and poorer social competence skills [19], compared to their typically developing peers. These social impairments in youth are associated with negative future outcomes, like increased school dropout and psychopathology [20,21]. Furthermore, pharmacological interventions, such as stimulant medication, are shown to not be effective in reducing social information processing impairments in youth with ADHD [22] and have been reported to exacerbate deficits in successfully processing information from social scenarios when compared to ADHD youth in a placebo group [23]. Therefore, examining other potential factors, such as sleep, that may be associated with improving social impairments in youth with ADHD is imperative.

## 2. Theory of Mind

An implicit form of ToM refers to a spontaneous detection of other’s mental states that is not deliberate or consciously inferred [24,25]. Implicit ToM presents early in childhood, with children correctly anticipating actions based on others’ mental states [24,25]. This can be seen in infants as young as 7 months, whose eye gazes correctly anticipate the goal-directed behaviors of others based on their personal beliefs [26]. Explicit ToM emerges around the age of 4 with the mastery of first order false belief tasks, signifying their awareness that individuals can hold beliefs that contrast with reality and their own beliefs [27,28,29]. ToM has been recognized as a pivotal milestone in social development for children across cultures [30,31,32]. Children develop more complex higher order ToM abilities as they age [33]. This includes successfully completing second order false-belief tasks, demonstrating an understanding of another person’s thoughts about a third person(s) [34]. For instance, a child demonstrating second-order false belief will predict that his/her friend will behave differently in a situation if their friend does not have the same new information as someone else (e.g., if the teacher changes the class location and the friend has not been informed, they will go to their regular class room, but the rest of the class who has been informed will go to the new room). 

Recognizing that two people can have different understandings of the same situation, and that someone’s underlying understanding might be different from what is apparent in reality [28], is implicated in several everyday tasks. These tasks include detecting deception and others’ emotions, and understanding the use of non-literal language (e.g., metaphors and sarcasm) [35], all of which are critical to social skills. Social skills are broadly defined as the ability to interact with others appropriately and effectively [36,37]. Such ToM skills, like detecting if someone is lying to you or understanding if someone is upset and why, are crucial to knowing how to appropriately engage in social situations. 

ToM ability is a predictor of social skills [3], as well as pro-social behavior [5,6,38] and emotional regulation [39,40]; whereas impairments in ToM are associated with higher rates of peer rejection [6,7], aggressive behavior [41,42], and diverse psychiatric disorders, including depression and anxiety [8,9,10], and neuro-developmental disorders, like autism [11]. 

ToM has cognitive and affective components [43,44,45]. Cognitive ToM refers to inferences about others’ beliefs and intentions, while affective ToM refers to inferences about others’ emotions and feelings. The distinction between the affective and cognitive components of ToM has been supported by studies showing performance can vary among ToM tasks, and impairments of one ToM component may not correspond to impairments of the other (see references [46,47,48,49,50,51] for examples). 

Neuroimaging studies support the existence of a ‘core neural network’ for ToM, which includes the medial prefrontal cortex and the bilateral posterior temporo-parietal junction. This network has been proposed because these neural areas are activated during all ToM tasks, irrespective of modality or stimuli, as they require thinking about the mental states of other persons [52]. Within this network, distinctions between affective and cognitive ToM have been made (for a comprehensive review, see refs [45,53]). Affective ToM engages the ventral medial prefrontal cortex, the inferior lateral frontal cortex, the ventral striatum, the ventral temporal pole, the ventral anterior cingulate cortex, the orbitofrontal cortex, and the amygdala [45,53]. Cognitive ToM recruits the dorsal medial prefrontal cortex, the dorsal lateral prefrontal cortex, the dorsal striatum, the dorsal temporal lobe, and the dorsal anterior cingulate cortex [45,53]. 

Tasks used to measure affective and cognitive ToM vary in types of stimuli and modality. They include situational narrative tasks and picture identification formats. The Reading the Mind in the Eyes Task [54,55] assesses affective ToM. It requires the identification of a word that best describes how a person is thinking or feeling based on photographs presented to them of the eye regions of human faces. It is dependent on emotional facial processing. The neural pathways involved in affective facial recognition and emotional information processing that include the amygdala and fusiform gyriare are implicated in the performance of this task [56,57]. Affective ToM tasks also require the ability to detect and regulate one’s own emotions. Emotional regulation refers to the intrinsic and extrinsic processes responsible for monitoring, evaluating, and modifying one’s own emotional reactions [58]. This ability enables a person to make inferences based on the feelings and emotions of others, while having an awareness of their own differing emotional state and regulating it appropriately in a situation. 

The Faux Pas Recognition Task [59] measures both cognitive and affective ToM abilities. It presents a story to a participant who is asked to determine if a character has said something socially inappropriate, a “faux pas” that would insult or hurt someone’s feelings (affective component), and asked if the “faux pas” was intended to hurt the listener’s feelings, and to determine the character’s intention to cause harm (the cognitive component). 

Although neural mechanisms underlying ToM have been characterized, behavioral and psychological factors that may influence or interact with these networks are still poorly understood. The most prevalent explanatory psychological mechanism put forth to account for ToM performance is executive functioning. 

### Executive Functioning and Theory of Mind

**Figure 1:**
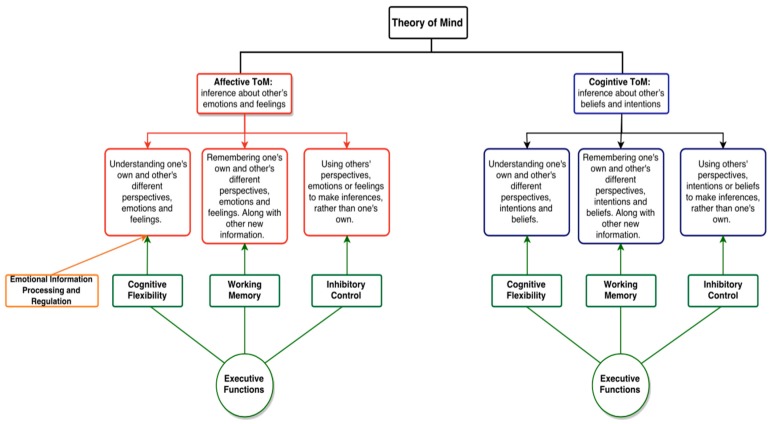
Flowchart demonstrating links between Theory of Mind (ToM) and executive functions (EF). Theory of mind is split into cognitive ToM, the ability to understand and make inferences about others’ beliefs and intentions; and affective ToM, the ability to understand and make inference about others’ emotions and feelings. Presented are the links found in literature [64,67,70] between affective and cognitive ToM in youth, with three EF subtypes: (1) cognitive flexibility; (2) working memory; and (3) inhibitory control. In addition to associations with EF, affective ToM is also associated with emotional information processing and regulation, needed to understand various feelings and emotions of others and oneself.

Executive functioning (EF) is an umbrella term that describes the cognitive processes that enable one to engage in deliberate, goal-directed thought and action [60,61]. The components of EF include: working memory, inhibitory control, and cognitive flexibility [60,61,62,63]. Working memory is the capacity to retain information in the short-term to guide future actions [60,61]. Inhibitory control is the ability to override prepotent responses, which involves being able to control one’s attention, behaviors, and/or emotions [61]. Cognitive flexibility (also known as ‘cognitive shifting’, ‘task shifting’, or ‘set-shifting’) refers to the ability to shift between tasks and adapt to new information [60]. Moderate to strong associations have been found between EF subcomponents and ToM in childhood [64]. The two constructs—EF and ToM—are reported to be dependent on the prefrontal cortex [65], and they both develop in a similar fashion from early to middle childhood [66,67,68]. EF and its subcomponents have been related to subcomponents of ToM (see Figure 1 for details) [67]. Inhibitory control is needed to inhibit salient knowledge of one’s current reality and one’s own emotions, beliefs, or intentions, in order to successfully respond to others’ mental states that are needed for both cognitive and affective ToM [69]. Cognitive flexibility is needed in order to shift between perspectives and mental states of others and oneself. Lastly, the capacity to actively retain multiple perspectives and information as one processes information requires working memory capacity. Collectively, EF abilities, including inhibitory control, cognitive flexibility, and working memory are simultaneously needed to successfully detect the mental states of others’ ToM. 

Although, the majority of research connecting EF and ToM has been conducted in pre-school children, there is evidence to suggest that this link extends into school age [70]. For instance, cognitive flexibility in school-age children predicts performance on social understanding tasks requiring affective and cognitive ToM abilities [71]. Cognitive flexibility, along with working memory, has also been shown to longitudinally predict affective and cognitive ToM ability on social scenario tasks in school aged children [70]. While very strong associations exist between inhibitory control and ToM in pre-school years [32,69,72], very little is known about this relationship as children develop.

## 3. Sleep

### 3.1. Sleep and Executive Functioning

According to the two-process model, sleep timing and duration is regulated by two distinct yet interacting biological processes, (1) the sleep-wake homeostasis (process S) and (2) the circadian rhythm (process C) [73]. A homeostatic sleep drive (i.e., the biological need for sleep) accumulates the longer a person is awake, causing pressure to fall asleep. The circadian rhythm controls the timing of sleep. It is an oscillatory rhythm that fluctuates with an approximate daily cycle of 24 h. The circadian rhythm is driven by an internal pacemaker, the biological clock, located in the superchiasmatic nucleus. External environmental stimuli known as zeitgerbers, which include light-dark cycles and temperature, influence circadian rhythms. Expert consensus recommends school children aged 6–12 years sleeping a duration of 9 to 11 h a night consistently to promote optimal health outcomes [74,75,76].

Insufficient and inadequate sleep are associated with poor executive functioning [77,78,79,80,81,82,83,84]. Insufficient sleep refers to getting less sleep than needed [85]. Inadequate sleep refers to poor-quality sleep, which includes low sleep efficiency, defined as the percentage of time in bed spent sleeping [86]. Neuroimaging studies reveal that sleep deprivation negatively disrupts the prefrontal cortex, a neural system central to EF [65,87]. Individuals with insomnia who experience poor sleep, characterized by frequent night awakenings, low sleep efficiency, troubles falling asleep at bedtime, and early morning awakenings [88,89], are shown to have altered connectivity in the frontostriatal networks [90]. Impaired frontostriatal connections are associated with EF deficits [91]. 

Shortened sleep duration and low sleep efficiency are associated with impaired inhibitory control abilities in youths and adults [92,93,94,95,96,97]. Behavioral tasks measuring the ability to suppress prepotent responses, known as inhibitory control, are negatively impacted by one to two nights of moderate sleep deprivation in adults [92,93]. After sleep deprivation an adult’s performance on the Go/No-Go Task, a computerized task whereby inhibitory control is measured by a participant’s ability to withhold responses to a known target, significantly deteriorates [92,93]. Similarly, six nights of moderate sleep deprivation, measured using actigraphy in typically developing school aged children and children diagnosed with ADHD, is shown to significantly impair their performance on the Continuous Performance Task (CPT) [94]. The CPT measures impulsivity and inhibitory control [98] by testing a participant’s ability to suppress their responses to a known target (i.e., the letter X), just like the Go/No-Go Task. One week of moderate sleep deprivation also alters brain activity measured by event-related potentials in school aged children compared to non-sleep deprived school aged children preforming the same inhibitory control task [96]. 

In addition to sleep deprivation, extending sleep duration by less than an hour has been found to improve inhibitory control abilities in children [99]. One study randomized school aged children into a sleep extension or sleep deprivation group [99]. They reported children in the sleep extension group, who slept an average of 35 min longer than their sleep-deprived peers, performed significantly better on the CPT. 

Typically developing children with low sleep efficiency, measured with actigraphy, are also shown to perform poorly on the CPT, compared to peers with higher sleep efficiency [95]. Taken together, these results provide a strong case for the impact sleep has on inhibitory control ability. 

The capacity to retain working memory is also affected by insufficient and poor-quality sleep in youth [99,100,101,102,103]. After going to bed one hour later than usual for four nights, school aged children’s working memory ability deteriorated compared to when they went to bed an hour earlier than their regular sleep schedule [103]. Two nights of actigraphy and one night of polysomnography (a gold standard biophysiological sleep measure, monitoring electroencephalography signals in the brain and other physiological movement during sleep) data of school aged children also revealed lower sleep duration was associated with poorer working memory ability compared to peers with higher sleep duration, as reported by their teachers on the revised Conners Teacher Rating Scale [100]. Sadeh et al. [99] found that extending school aged children’s sleep duration, monitored by actigraphy, significantly improved their performance of the Digital Span task. Low sleep efficiency in children, measured for 72 h using actigraphy, is also shown to be associated with poor auditory and visual working memory performance on the n-back task [102]. Overall, evidence to date demonstrates short sleep duration and low sleep efficiency significantly impairs working memory ability. 

Cognitive flexibility in youth is shown to be affected by sleep duration [104]; however, limited research exists and its relation to sleep efficiency is unknown. In adolescents, two weeks of extending sleep duration 5 min a night, measured using actigraphy, was found to have positive performance effects on the Divided Attention task [104]. The task requires participants to actively shift their attention between an initial stimulus while simultaneously processing others in order to correctly detect different target sequences corresponding to cognitive flexibility. This demonstrates a link between longer sleep duration and better cognitive flexibility performance in youth. 

**Figure 2:**
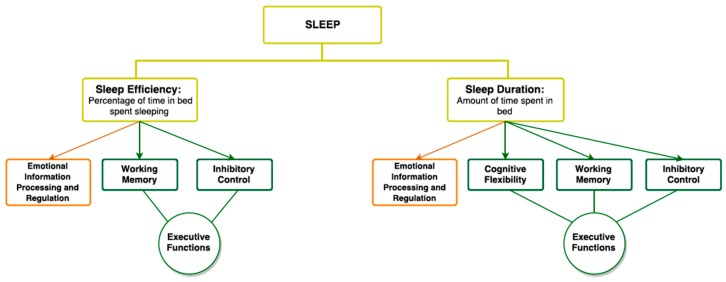
Conceptual framework demonstrating links between ToM and EF. The present chart demonstrates the links found in the literature between two sleep variables, the sleep efficiency (percentage of time in bed spent sleeping) and the sleep duration (amount of time spent in bed sleeping), and EF subtypes. Sleep duration is associated with cognitive flexibility [104], working memory [99,100,103], and inhibitory control [92,93,94,99]. Sleep efficiency is associated with working memory [102] and inhibitory control [97]. Both sleep duration and efficiency have been shown to be associated with emotional information processing and regulation [57,105,108,109].

In all, a great deal of evidence directly links sleep duration and efficiency with all three EF subcomponents: working memory, inhibitory control, and cognitive flexibility (see Figure 2 for an overview). 

### 3.2. Sleep and Emotional Information Processing

Sleep has been shown to be associated with emotional information processing and regulation, key processes needed for the proper function of affective ToM. 

Sleep deprivation is associated with altered brain activation when viewing negative salient emotional stimuli [57]. Those who are sleep-deprived experience a greater magnitude of amygdala activation when seeing aversive stimuli. This demonstrates that shortened sleep duration intensifies emotional reactivity, which challenges the ability to successfully regulate ones’ own emotions. Also, compared to non-sleep deprived individuals, sleep-deprived participants who viewed emotional stimuli on pictures showed reduced functional connectivity between the amygdala and medial prefrontal cortex, an area found to be involved in the top-down modulation of emotional processing and responses [57].

Additionally, sleep deprivation elevates the activation of the ventral anterior cingulate cortex [105]. Increased activation of the ventral anterior cingulate cortex is linked to detecting and regulating emotions in school aged children [106].

Shorter sleep duration and poor-sleep quality has been associated with reduced emotional information processing abilities in both adults and youth [103,107,108]. This includes an impaired ability to match emotions to faces [109], a ToM deficit. A 2011 study [109] found that elevated night awakenings and decreased sleep efficiency predicted poor performance in identifying simple facial emotions (e.g., happy and sad) in school aged children. 

Furthermore, neuroimaging studies have found longer sleep duration and sleep credit (sleeping more than the minimal duration needed to avoid impairment) are related to greater grey matter volume of the medial frontal and orbitofrontal cortex regions, and are also associated with higher emotional intelligence [110,111]. Emotional intelligence includes the ability to respond flexibly to changing emotional information and understanding others’ emotions [110,111]. 

All of these emotional processing and regulating abilities disrupted by poor-sleep duration and efficiency are directly needed for successful affective ToM.

Overall, shorter sleep duration and lower sleep efficiency are associated with an impaired ability to regulate one’s own emotions and process emotional information, which are central to successfully understanding other people’s emotional mental states.

## 4. Sleep and ToM: Are They Associated?

A large body of evidence has shown that poor sleep is associated with impaired EF and with poor emotional information processing abilities. Both of these impairments correspond to ToM deficits. Neural networks disrupted by poor sleep also correspond to brain areas involved in the affective and cognitive ToM network. 

Given EF is strongly entrenched in the development of ToM, and poor sleep impairs EF ability, we propose that poor sleep may be associated with poor cognitive ToM. Based on evidence demonstrating that poor sleep worsens the ability to process emotional information (such as emotions on faces), we propose that poor sleep will be associated with poor affective ToM. 

Future research should examine the relationship between sleep and ToM to determine if any casual associations can be identified. This information can then eventually be applied to forming the basis of developing innovative approaches to support children with dysfunctional ToM and improve their future social cognitive development.

## 5. Attention Deficit/Hyperactivity Disorder

### 5.1. ADHD, EF, and Social Functioning 

Attention-Deficit/Hyperactivity Disorder is a prevalent developmental disorder that affects children and adults. It is defined by atypical levels of inattention, impulsivity, and hyperactivity, and occurs in 5% of school aged children [14]. Children diagnosed with ADHD are characterized as having impaired EF [112,113]. They exhibit significant impairment in all subcomponents of EF, including cognitive flexibility and working memory [114]; however, the most robust deficits are seen in the inhibitory control [114]. Brain regions corresponding to EF performance, like the prefrontal cortex, are shown to activate atypically in youth diagnosed with ADHD [115,116,117].

Literature on ADHD has mostly focused on its impact on cognition and academic performance [118,119,120], reading [121], or organizational skills needed for academic success [122,123], but has sparsely focused on social cognition. This is despite well-documented reports of youth with ADHD having severe social impairments [124,125]. Children diagnosed with ADHD are more often rejected by their peers and have fewer friends compared to typically developing children [16,126]. In fact, within a few hours or even minutes of a social interaction with unfamiliar peers, youth with ADHD are often disliked and make negative impressions [17,127,128]. Additionally, children with ADHD have been found to express impaired empathy [18], are socially intrusive [125], have fewer reciprocal dyadic friendships [129], and are rated as less socially competent compared to typically developing peers by parents and teachers [19] relative to non-ADHD youth. Although youth with ADHD struggle with interpersonal and social competence skills, they inaccurately perceive their own abilities, overestimating their social competence when self-evaluating [130]. Such interpersonal and social skill impairments, like peer rejection, are troubling, as they are associated with negative future outcomes including dropping out of school, substance abuse, delinquency, and higher rates of psychopathology [20,21].

### 5.2. ADHD and ToM 

Studies have found ToM deficits are present in youth diagnosed with ADHD [131,132]. Research findings indicate youth with ADHD exhibit emotional regulation deficits [133] and impaired affective information processing [134,135] needed for successful ToM. In comparison to typically developing peers, youth with ADHD show reduced amygdala activation when processing fearful expressions [135] and perform poorly on social decision problems requiring the processing of others’ facial emotions to appropriately solve a social problem [136]. Additionally, youth with ADHD have difficulties detecting positive and negative cues in social stories, including interpreting other people’s intentions [137]. One study [131] found children with ADHD performed worse than typically developing children on the Reading the Mind in the Eyes Task and the Faux Pas Recognition Task, testing affective and cognitive ToM. Poor inhibitory control and attention deficits predicted performance on the Faux Pas Recognition and Reading the Mind in the Eyes tasks, respectively, in youth with ADHD. 

### 5.3. ADHD and Sleep

Sleep problems are common in children with ADHD [12], with parents reporting 2- to 3-times higher prevalence of sleep disturbances compared to normal controls [13]. Sleep disorders that have been reported to be more prevalent in children with ADHD compared to their typically developing peers include Periodic Leg Movement Disorder and Restless Leg Syndrome (PLMS), and sleep disordered breathing [138,139,140]. Poor sleep negatively impacts EFs, which are already disrupted in children with ADHD. Hence, these sleep issues can contribute to exacerbating existing cognitive, emotional, and social deficits in youth with ADHD [141].

The novel link proposed between sleep and ToM in section 4 can provide insight into the associations between sleep issues and social deficits observed in youth with ADHD. Current literature has associated ADHD and ToM with deficits in EF. Inadequate and insufficient sleep negatively affect EF, and sleep issues are found to be prevalent in youth diagnosed with ADHD. As youth with ADHD exhibit deficits with interpersonal skills and social cognitive functioning, including impaired ToM, sleep may be a key factor contributing to these deficits by impairing EF and emotional information processing. Future research is warranted to examine the interplay between sleep, ToM, and socio-emotional cognition in children with and without ADHD. If casual associations are uncovered, this information can then eventually be applied to forming the basis of developing innovative approaches in the treatment of social dysfunction in youth. 

## 6. Conclusions

This paper has presented substantial evidence that poor and inadequate sleep may be associated with ToM impairments in youth with and without ADHD. Sleep deprivation causes impairments in EF and emotional information processing, both of which are associated with poor ToM ability. EF deficits are a core impairment in youth with ADHD, who also experience greater sleep issues compared to their typically developing peers. Since youth with ADHD are known to experience social dysfunction, which includes ToM deficits, sleep may be a contributing factor by impairing EF. Therefore, examining the role of sleep in relation to the social deficits that characterize youth with ADHD may provide helpful insights into understanding and treating the social impairments identified.
